# Primary hepatic carcinoid; a diagnostic dilemma: a case report

**DOI:** 10.1186/1757-1626-1-314

**Published:** 2008-11-17

**Authors:** Zisis Touloumis, Spiros G Delis, Charikleia Triantopoulou, Nikoletta Giannakou, Costas Avgerinos, Christos Dervenis

**Affiliations:** 1 Liver Surgical Unit, First Department of Surgery, "Agia Olga" General Hospital, 3-5 Agias Olgas street, 14233 N. Ionia, Athens, Greece; 2 Department of Computed Tomography, "Agia Olga" General Hospital, 3-5 Agias Olgas street, 14233 N. Ionia, Athens, Greece; 3 Department of Pathology, "Agia Olga" General Hospital, 3-5 Agias Olgas street, 14233 N. Ionia, Athens, Greece

## Abstract

**Introduction:**

Primary hepatic carcinoid tumours (PHCTs) are extremely rare neuroendocrine neoplasms. Only 58 cases have been reported in the literature and less than 10 cases were functional.

**Case presentation:**

We present a case of a 65 years old, Caucasian female with a large unresectable primary hepatic carcinoid tumor secreting 5-hydroxyindoleacetic acid (5-HIAA), presented with flushing and diarrhoea and treated with trans-catheter arterial embolization (TACE) and subsequent administration of lanreotide (long acting somatostatin analogue).

**Conclusion:**

The diagnosis of PHCTs is difficult due to their common radiologic characteristics with other liver lesions. Their diagnosis is based on the exclusion of other sites of disease and the histologic confirmation. Although the mainstay of treatment when is technically feasible is surgical resection with optimal 5-year survival and low recurrence rate, in cases of unresectable disease palliation with combination of TACE and administration of somatostatin analogues have good results in controlling the disease and the patients symptoms.

## Introduction

Carcinoid tumours are of neuroendocrine origin, well-differentiated low-grade malignant neoplasms. They follow an indolent course in comparison with other malignant neuroendocrine carcinomas, which display varying degrees of pleomorphism, mitotic activity, vascular invasion, and necrosis [1]. Nonetheless, carcinoid tumours can be quite unpredictable and may cause carcinoid syndrome [2]. They occur most frequently in the gastrointestinal tract and the respiratory system and they frequently metastasize to the liver [3-5]. Primary hepatic carcinoid tumours (PHCTs) are rare with only 58 cases described in the literature since the first report in 1958 by Edmondson [6]. The rarity of PHCT makes it difficult for clinicians to suspect and diagnose it precisely. Although PHCT shows a slow growth, surgical resection is the treatment of choice. In case of unresectability due to anatomical reasons TACE and long acting somatostatin analogues (SSAs) can stabilize the disease and provide long term palliation.

## Case presentation

**Figure 1:**
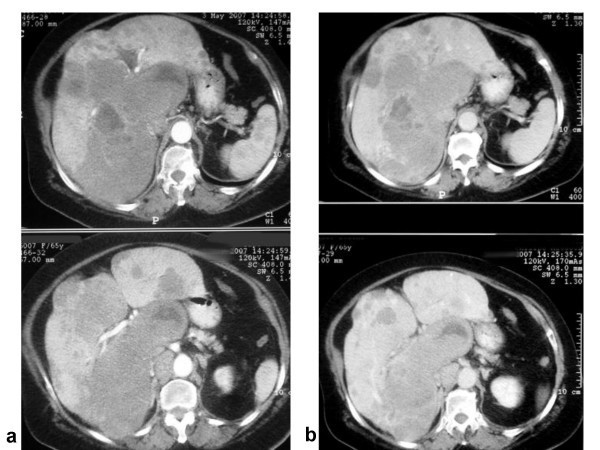
Contrast enhanced CT, arterial (a) and portal venous phases (b): many lesions are demonstrated in both liver lobes showing progressive contrast enhancement.

**Figure 2:**
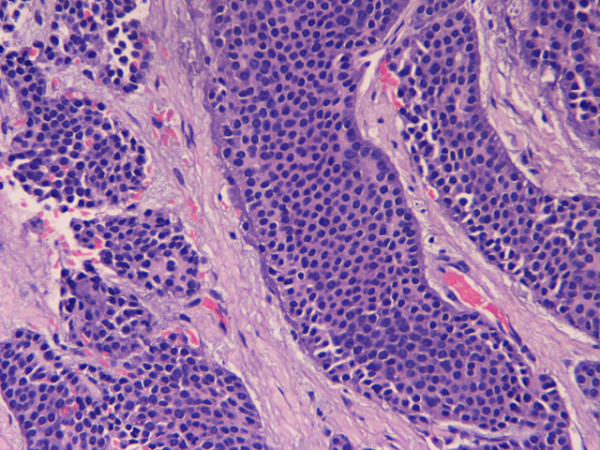
Liver carcinoid. Typical morphology of low-grade endocrine tumor showing insulinar arrangement of medium-sized cells with round nuclei and peripheral palisading. The stroma is vascular and fibrotic. E/H × 400.

**Figure 3:**
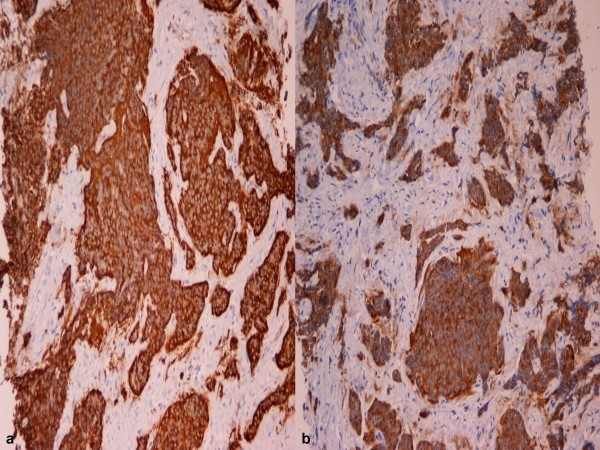
(a) Strong immunopositivity for chromogranin A. PAP × 200. (b) Strong immunopositivity for synaptophysin. PAP × 200.

A 65 years old, Caucasian female patient presented on admission to our Liver Surgical Unit with a history of flushing, diarrhoea, vomiting and low grade fever. Ultrasound (US) demonstrated multiple hypoechoic lesions in both liver lobes. Computed Tomography (CT) revealed heterogeneous hypodense lesions invading most of the hepatic parenchyma. The lesions showed mild gradual enhancement during the arterial and portal phases, while cystic areas were also demonstrated. The larger of the lesions showed extrahepatic extension compressing and dislocating the duodenum. No vascular invasion was noted (fig. 1a, b). A 24 h-urine 5-hydroxyindoleacetic acid (5-HIAA) level was high (36,4 mg/day – normal range: 1–6 mg/day); the tumor markers CEA and Ca 19-9 were within normal range. The patient underwent a CT-guided biopsy of the larger lesion and the histology revealed a well differentiated tumor, composed of solid (insular) nests or ribbons of monomorphic, medium-sized cells, with variably eosinophilic neoplasm and round nuclei. Peripheral palisading was also noted in some solid nests (fig. 2) with no tumor necrosis. The morphologic features of the tumor were compatible with a neuroendocrine tumor. The diagnosis was confirmed by immunohistochemistry. The neoplastic cells were proven to be positive for the endocrine markers Chromogranin A, Synaptophysin, NSE and CD56, in addition to Cytokeratins Cam 5.2, 5/6 and 19 (fig. 3a, b). No immunohistochemical findings were suggestive for carcinomatous (metastatic or primary) or metastatic melanocytic growth identified.

In addition to these diagnostic modalities the patient underwent an abdominal and chest CT scan along with abdominal MRI, mammography and an upper and lower GI endoscopy which didn't reveal the primary. A total body octreoscan with In^111^, a MIBG scan and a [^11^C] 5HTP-PET showed intense uptake of the radiotracer exclusively in the liver. Based on the biochemical and thorough radiological investigation, the diagnosis of PHCT was made with certainty. Due to the extended liver invasion, resection wasn't feasible and palliation was performed with TACE and subsequent administration of lanreotide (long acting somatostatin analogue) 30 mg/28 days subcutaneous for 22 months. TACE included streptozotocin 1.5 g/m^2 ^dissolved in 10 ml of normal saline (0,9%) combined with 10 ml of lipiodol. The emulsion was injected into the branches of the hepatic artery distal to the origin of gastro-duodenal artery. Two years later the patient is free of symptoms with no radiologic disease progression.

## Discussion

Carcinoid tumors are rare, slowly growing neuroendocrine neoplasms that account for only 2% of all gastrointestinal tumors [7]. PHCTs are extremely rare neoplasms reported infrequently. PHCTs are detected incidentally and occur usually in patients older than 50 years and slightly more often in females. They show a slow growth and in most cases they are non-functional [5,8].

Their histogenesis hasn't been clarified. It is hypothesized that they arise from scattered neuroendocrine cells in the intrahepatic biliary epithelium. Another possible explanation is that chronic inflammation in the biliary system may predispose to the development of neuroendocrine tumor by initiating intestinal metaplasia or even that these tumors originate from heterotopic pancreatic or adrenal tissue, mainly in the central part or perihilar areas of the liver, where heterotopic pancreatic tissue is most often located. However, the clinician must be aware of the possibility that an apparent PHCT might be a metastatic one of a primary tumor that remains obscure despite an extensive assessment.

Diagnosis of primary hepatic carcinoid tumors is difficult as the radiologic appearance on ultrasound, CT, and MRI can mimic hepatocellular carcinoma, cholangiocarcinoma and metastases of unknown origin. A solid mass with cystic areas and hyperechoic or mixed pattern is a common finding detected by ultrasound. In addition calcifications are sometimes demonstrated along the margin of the cysts. Of note they are markedly hypervascular on contrast-enhanced US with significant enhancement [9]. Contrast enhanced CT permits a comprehensive dynamic study of neuroendocrine liver lesions. Small carcinoids are enhanced homogeneously in the arterial phase, while large tumors usually show a peripheral pattern of enhancement. In late phases most lesions evolve into low density masses, but progressive enhancement has also been described and presumably corresponds to proliferative fibrous tissue inside the lesion [10]. On MRI primary carcinoids usually present with low intensity in T1-w and high intensity on T2 images.

Intra-lesion solid components are enhanced in the early arterial phase in a similar fashion with hepatocellular carcinoma. After the administration of a specific liver contrast (super paramagnetic iron oxide) the extent of the tumor is more clearly delineated due to the decrease of normal liver parenchyma signal intensity [11]. On angiography the characteristic hypervascularity is evident with or without staining. Nowadays with the evolution of CT or MR angiography, interventional diagnostic studies could not be considered as first line examinations. However, we can not depend on cross-sectional imaging modalities for accurate diagnosis, due to overlapping imaging features with other hypervascular tumors like HCC or adenoma. Although specific liver contrast agents are helpful in the diagnosis of PHCTs, liver biopsy is warranted to exclude other more common lesions. Tumor cells usually stain positive with chromogranin, synaptophysin and neuron-specific enolase. Octreoscan is a valuable tool, permitting not only accurate diagnosis of neuroendocrine tumors but also excluding small metastatic deposits in bones or lymph nodes, which CT or MRI fail to detect [12]. PET scan has been recognized as a useful tool in visualization of neuroendocrine tumors (NET) that lack somatostatin receptor type 2 and are negative on somatostatin receptor scintigraphy (SRS). In our case combined with SRS and MIBG, [^11^C] 5HTP-PET demonstrated the primary nature of liver carcinoid.

It appears that most PHCTs are functionally silent. Presentation with abdominal distension and discomfort is most common, and carcinoid syndrome is rare although is documented in our case. This is the second case reported in the literature of carcinoid syndrome related to PHCT and proved by abnormal HIAA urine levels.

The mainstay of treatment of primary hepatic carcinoid tumor is liver resection. In the literature up to 85% of patients with PHCT have resectable disease [13]. When PHCT is confined to a single lobe, surgical resection should be taken into consideration, and a postoperative 5-year survival rate of 74% has been reported, with a recurrence rate of 18% [8]. Whether bilateral non resectable lesions exist, palliative cyto-reductive surgery in combination with TACE might be effective [14]. TACE has been reported to be effective in terms of temporary control of the disease or prolongation of free disease survival in selective patients [15]. The role of somatostatin analogues is well defined regarding stabilization of the metastatic neuroendocrine disease and control of symptoms. However the efficacy of SSAs as a treatment option for PHCT is not well defined. In our single case the combination of TACE with lanreotide was helpful to offer long term palliation.

## Conclusion

PHCTs represent a rare entity difficult to diagnose by the conventional radiology studies. Exclusion of another primary and liver biopsy is strongly warranted. The natural history of these lesions is long and a median survival of several years has been reported for patients who have been treated by resection. TACE with long acting somatostatin analogues might be helpful to offer long term palliation.

## Abbreviations

PHCTs: Primary hepatic carcinoid tumours; 5-HIAA: 5-hydroxyindoleacetic acid; TACE: trans-catheter arterial embolization; SSAs: long acting somatostatin analogues; SRS: somatostatin receptor scintigraphy.

## Consent

Written informed consent was obtained from the patient for publication of this case report and accompanying images. A copy of the written consent is available for review by the Editor-in-Chief of this journal.

## Competing interests

The authors declare that they have no competing interests.

## Authors' contributions

ZT analyzed and interpreted the patient data and was a major contributor in writing the manuscript; SD analyzed and interpreted the patient data regarding and was a major contributor in writing the manuscript; CT performed the CT investigation of the patient and contributed in writing the manuscript; NG performed the histological examination of the liver biopsies and provided the histological images; CA contributed in writing and revising the manuscript; CD contributed in writing and revising the manuscript.

All authors read and approved the final manuscript.

## References

[B1] Staren ED, Gould VE, Warren WH, Wool NL, Bines S, Baker J, Bonomi P, Roseman DL, Economou SG (1988). Neuro-endocrine carcinomas of the colon and rectum: a clinicopathologic evaluation. Surgery.

[B2] Knox CD, Anderson CD, Lamps LW, Adkins RB, Pinson CW (2003). Long-term survival after resection for primary hepatic carcinoid tumor. Annals Surg Oncol.

[B3] Moertel CG (1987). Karnofsky memorial lecture: an odyssey in the land of small tumors. J Clin Oncol.

[B4] Shebani KO, Souba WW, Finkelstein DM, Stark PC, Elgadi KM, Tanabe KK, Ott MJ (1999). Prognosis and survival in patients with gastrointestinal tract carcinoid tumors. Ann Surg.

[B5] Norheim I, Oberg K, Theodorsson-Norheim E, Lindgren PG, Lundqvist G, Magnusson A, Wide L, Wilander E (1987). Malignant carcinoid tumors: an analysis of 103 patients with regard to tumor localization, hormone production, and survival. Ann Surg.

[B6] Edmondson HA, Edmodson HA (1958). Carcinoid tumor. Tumors of the liver and intrahepatic bile ducts.

[B7] Wallace S, Ajani JA, Charnsangavej C, DuBrow R, Yang DJ, Chuang VP (1996). Carcinoid tumors: imaging procedures and interventional radiology. World J Surg.

[B8] Miura K, Shirasawa H (1988). Primary carcinoid tumor of the liver. Am J Clin Pathol.

[B9] Komatsuda T, Ishida H, Furukawa K, Miyauchi T, Heianna J (2005). Primary carcinoid tumor of the liver: report of a case with an emphasis in contrast-enhanced ultrasonographic findings. J Clin Ultrasound.

[B10] Takayasu K, Muramatsu Y, Sakamoto M, Mizuguchi Y, Moriyama N, Wakao F, Kosuge T, Takayama T, Hirohashi S (1992). Findings in primary hepatic carcinoid tumor: US, CT. MRI and angiography. J Comput Assist Tomogr.

[B11] Kehagias D, Moulopoulos L, Smirniotis V, Pafiti A, Ispanopoulos S, Vlahos L (1999). Imaging findings in primary carcinoid tumor of the liver with gastrin production. BJR.

[B12] Yüksel M, Eziddin S, Ladwein E, Haas S, Biersack HJ (2005). 111In-pentetreotide and 123I-MIBG for detection and resection of lymph node metastases of a carcinoid not visualized by CT, MRI or FDG-PET. Ann Nucl Med.

[B13] Fenwick SW, Wyatt JI, Toogood GJ, Lodge JP (2004). Hepatic resection and transplantation for primary carcinoid tumors of the liver. Annals of Surg.

[B14] Sano K, Kosuge T, Yamamoto J, Shimada K, Takayama T, Yamasaki S, Makuuchi M (1999). Primary hepatic carcinoid tumors confirmed with long term follow up after resection. Hepatogastroenterology.

[B15] Mehta DC, Warner RR, Parnes I, Weiss M (1996). An 18-year follow-up of primary hepatic carcinoid with carcinoid syndrome. J Clin Gastroenterol.

